# Step by step approach to rare breast lesions containing spindle cells

**DOI:** 10.1186/s40064-015-1480-y

**Published:** 2015-11-05

**Authors:** Betül Ünal, Gülgün Erdoğan, Fatma Şeyda Karaveli

**Affiliations:** Department of Pathology, Faculty of Medicine, Akdeniz University, 07070 Antalya, Turkey

**Keywords:** Spindle cell, Breast, Differential diagnosis, Immunohistochemistry, molecular changes

## Abstract

Differential diagnosis of spindle cell lesions of breast is challenging for certain reasons. The most important reason is the presence of cytological atypia and mitosis in all three conditions: reactive, benign, and malignant. Patients diagnosed with benign and malignant tumor/tumor-like lesions that had spindle cell components following the histopathological examination were included in the study. The patients’ medical records were accessed to obtain the clinical history, follow-up notes, and radiological findings. Following histopathological, immunohistochemical, and clinical evaluations, the patients were diagnosed as follows: pseudoangiomatous stromal hyperplasia (PASH), bilateral desmoid-type fibromatosis (FM), adenomyoepithelioma (AME), myofibroblastoma (MFB), malignant phyllodes tumor (MF), high-grade AS, post-chemotherapy osteosarcoma (OS) + Paget’s disease, and metaplastic carcinoma (MC). An algorithmic approach should be used in the diagnosis; cellular structure, presence and grade of atypia, growth pattern, mitotic activity, immunohistochemical staining, and clinical and radiological features should be evaluated together. Detection of some molecular changes can be useful in differential diagnosis.

## Background

Spindle cell lesions of the breast are rare entities; however, the differential diagnosis is challenging for certain reasons (Länger et al. 2014). The most important reason is the presence of cytological atypia and mitosis in all three conditions: reactive, benign, and malignant. In addition, clinical, radiological, and immunohistochemical similarities can be seen in these lesions. Varma and Shin (2013) suggested an algorithmic approach for the differential diagnosis, and stated that the following parameters should be definitely evaluated: (1) cellular structures, (2) presence and degree of atypia, (3) growth pattern, (4) mitotic activity, and (5) clinical and radiological features (Varma and Shin 2013; Al-Nafussi 1999).

The size of the histopathologically examined specimen is particularly important for biphasic tumors. For instance, it is possible to see only one epithelial and stromal component in the core biopsy materials. Thus, this condition leads to difficulty in the diagnosis, or misdiagnosis.

When approaching lesions that contain only spindle cells, attention should be given primarily to cytomorphology. If the cells show atypia, the following conditions should be considered in the differential diagnosis: spindle cell metaplastic carcinoma, adenomyoepithelioma, adeno sarcoma (AS), osteosarcoma, myofibroblastic sarcoma, other primary breast sarcomas, and metastasis. If the cells do not show signs of prominent atypia, the following conditions should be initially considered in the differential diagnosis: fibromatosis, granulation tissue, pseudoangiomatous stromal hyperplasia (PASH), low-grade AS, myofibroblastoma, inflammatory myofibroblastic tumor, nodule with spindle cells, lipoma with spindle cells, schwannoma, and neurofibromas (Tan and Ellis 2013; Lakhani et al. 2012; Tavassoli and Devilee 2003; Stolnicu et al. 2015).

In some cases, epithelial cells can be seen, in addition to spindle cells. In these cases, spindle cell metaplastic carcinoma and fibro epithelial lesions (fibro adenoma and phyllodes tumor) should be considered first. If glandular structures are seen with spindle cells, adenomyoepithelial tumors should be considered in the differential diagnosis.

In the present study, we evaluated cases of spindle cell lesions of the breast with different diagnoses (reactive, benign, and malignant). We discussed these extremely rare lesions together with their differential diagnosis, to better understand their clinical findings, pathological findings, and immunohistochemical profiles.

## Methods

Seven patients who underwent breast surgery at Akdeniz University Faculty of Medicine between 2007 and 2014, and who were diagnosed with benign and malignant tumor/tumor-like lesions that had spindle cell components following the histopathological examination were included in the study. The patients’ medical records were accessed to obtain the clinical history, follow-up notes, and radiological findings. All protocols adhered to the tenets of the Declaration of Helsinki and were approved by the institutional review board of the Akdeniz University Medical Faculty. Written formed consents were acquired from all participating patients.

We accessed the slides (stained with hematoxylin-eosin; immunohistochemically examined) of all patients from the archive, and all slides were re-evaluated by two pathologists (B.Ü. and G.E.). Where necessary, new serial sections were obtained from paraffin-embedded tissues, and microscopic examinations were performed after additional immunohistochemical staining. For the differential diagnosis, various parameters were used to evaluate each patient. These included: 1-macroscopic examination, 2-microscopic examination, 2a-cellular component, 2b-presence and degree of atypia, 2c-growth pattern, 2d-mitotic activity, 2e-presence of other comorbid components, 3-immunohistochemical analysis, and 4-clinical and radiological findings.

For the differential diagnosis of spindle cell lesions of the breast, the diagnostic features described in the WHO classification of breast lesions were used (Lakhani et al. 2012).

## Results

The mean age of eight female patients was 46.2 (min: 21–max: 74) years. Following histopathological, immunohistochemical, and clinical evaluations, the patients were diagnosed as follows: pseudoangiomatous stromal hyperplasia (PASH), bilateral desmoid-type fibromatosis (FM), myofibroblastoma (MFB), malignant phyllodes tumor (MF), high-grade angiosarcoma (AS), post-chemotherapy osteosarcoma (OS) + Paget’s disease, and metaplastic carcinoma (MC). Among these patients, the patient diagnosed with MC was consultation material and we could not access the clinical and macroscopic examination results. The other seven patients were admitted to the clinic with complaints of palpable mass, and only the patient with AS had complaints of pain. The patient with bilateral FM was admitted with depression in both nipple. The patients with OS were admitted with masses of the breast, redness in the papilla, and eczematous appearance. In the patient with MF, a mass with rapid growth in the last 10 months was described. Among patients who underwent mammography, MFB was classified as category 3, whereas AME, MF, and AS were classified as category 4. The mean diameter of the tumors was 6.03 (min: 1.6–max: 25) cm, and MF had the highest mean diameter (25 cm). The patients underwent FNAB, core biopsy, excisional biopsy, lumpectomy, simple and radical mastectomy, and positive margins were seen in the cases of patients with FM and AS. For other tumors, lesions were negative in the surgical borders (Table 1). The macroscopic examination showed that the tumor section faces of PASH, MF, and AME had a yellow-white color, and these were well-circumscribed and solid nodular lesions. FM was benign, but showed an infiltrative dissemination pattern; the lesion had an oyster white color and fibrotic appearance. MF showed typical macroscopic findings, including pink-white section face, bulging, curved clefts, and a whorled pattern. In the OS case, an infiltrative pattern, bone and cartilage tissue, grey-white section face, and hard and soft areas were observed (Table 2). In the immunohistochemical examination, staining procedures, which were required for the differential diagnosis, were performed, and these findings are summarized in Table 3. In addition, an evaluation of patients’ histories showed that patient with OS received 17 cycles chemotherapy in 2010–2011 for a period of 1 year.

**Table 1:** Clinicopathological characteristics of patients

	PASH	FM	MFB	AME	MF	MC	AS	OS
Age	31	21	57	65	57	74	28	36
Localization	Left	Bilateral	Right	Right	Right	NA	Right	Left
Complaint	Mass	Nipple depression	Mass	Mass, pain	Fast-growing mass in last 10 months	NA	Pain	Mass, eczematous nipple
Mammography	No	No	Cat 3	Cat 4A	Cat 4	NA	Cat 4	No
Procedure	ecs.bx	ecs.bx, bilateral	ecs.bx	cor bx, lumpectomy	cor bx, simple mastectomy	NA	FNAB, ecs.bx	cor bx, lumpectomy, MRM
Tumor size	2,5 cm	Right 4 cm, left 4.5 cm	1.6 cm	1.6 cm	25 cm	NA	2 cm	5 cm
Margins	(−)	Right and left(+)	(−)	(−)	(−)	NA	(+)	(−)

**Table 2:** Summary of macroscopic findings

	PASH	FM	MFB	AME	MF	MC	AS	OS
Macroscopic findings	Yellow-white Expansile Well-circumscribed Solid	Off-white Infiltrative Firm Fibrotic	Yellow-white Nodular Well-circumscribed	White-tan Well-circumscribed Nodular	White-tan-pink Bulging Well-circumscribed Curved clefts Whorled pattern	Yellow-white Infiltrative	Brown-tan Infiltrative Necrosis Haemorraghic	Gray-white Infiltrative Firm and soft areas Bone, cartilage Eczematous nipple

**Table 3:** Immunohistochemical analysis results

	PASH	FM	MFB	AME	MF	MC	AS	OS
IHC findings	CD34, Vimentin stromal (+), F8, CD31 (−)	Desmin, CK14, PS2, ER, PR, Bcatenin, S100 (−), Actin diffuse, bcl-2, CD34 focal (+)	CK, S100, E-cad (−) Vim, SMA, Desmin, CD34 diffuse (+), Calponin focal (+)	MYE cells; Calponin P63 CK14 (+) Epithelial cells; HMWK (+)	KI67 %25	CD34 S100 Desmin Actin (−) PanCK focal (+)	PanCK SMA (−) F8 CD34 Vimentin (+)	Vimentin (+) CD34 D2-40 F8 Actin Desmin PanCK YMAK CK5/6 CK14 ERPR cerbB2 EMA S100 (−) KI67 %75 Paget cells; CK7,cerbB2 (+)

### Microscopic evaluation

**Fig. 1:**
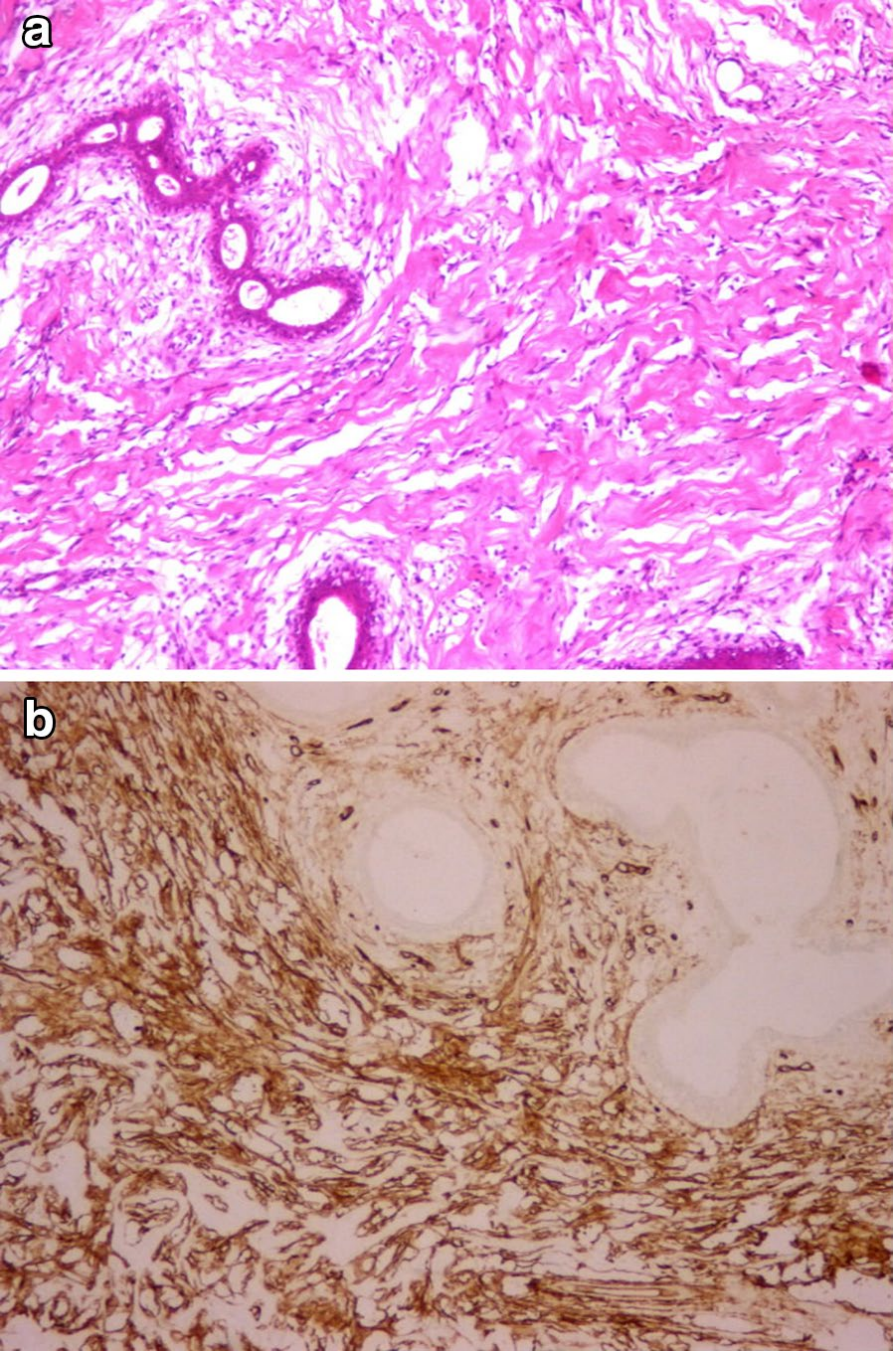
Myofibroblastic cells and anastomosing vascular spaces were seen between the breast lobules (**a** H&Ex100), CD34 positivity (**b** ×200)

PASH: Stromal myofibroblastic cells and vascular spaces that were covered with spindle cells and were anastomosing to each other were seen between the breast lobules (Fig. 1). Mitosis and atypia were not observed. Necrosis and infiltrative boundaries were not seen; the lesion appeared well-circumscribed.

**Fig. 2:**
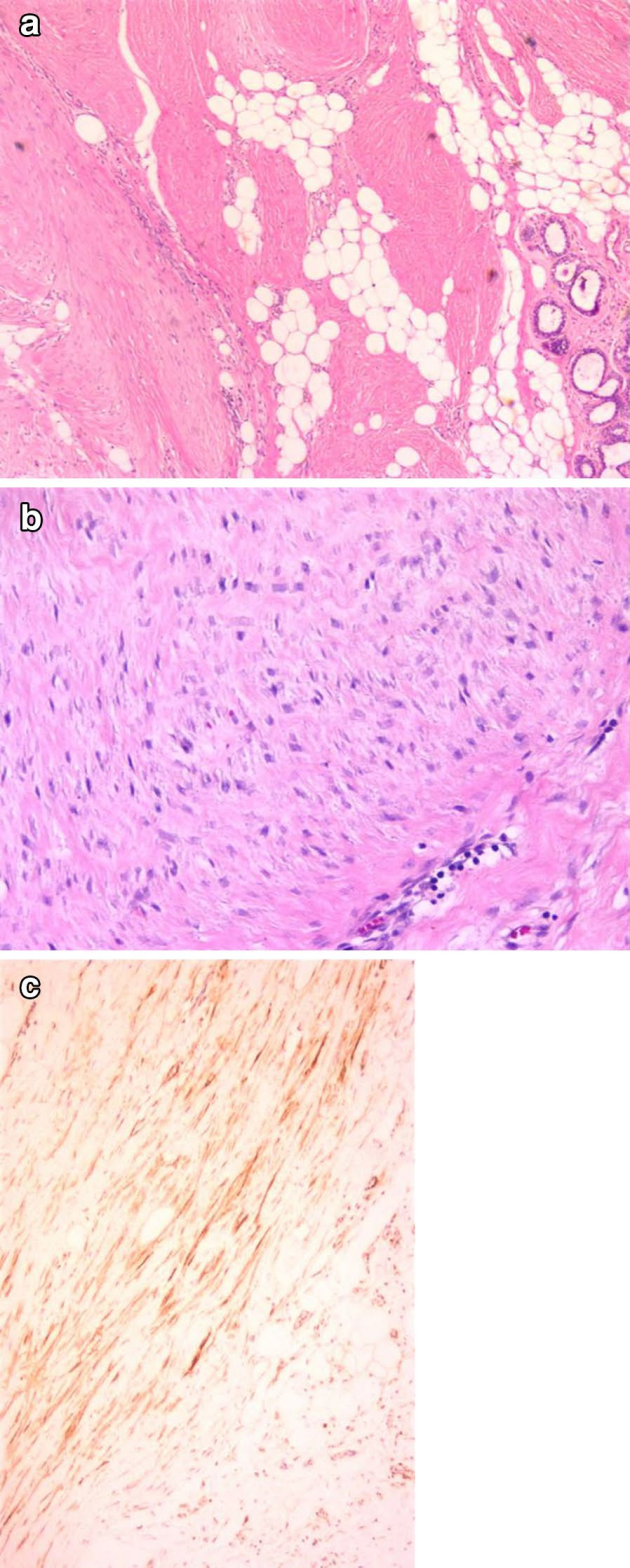
Spindle cells that formed fascicules in the collagenous stroma (**a** H&Ex50; **b** H&Ex200), actin positivity (**c** ×100)

MFB: Consisted of fibroblasts and myofibroblasts; benign, well-circumscribed tumoral lesion (Fig. 3).

FM: The lesion consisted of spindle cells that formed fascicules in the collagenous stroma, and showed infiltrative dissemination to the neighboring areas. Prominent atypia and mitosis were not observed (Fig. 2).

**Fig. 3:**
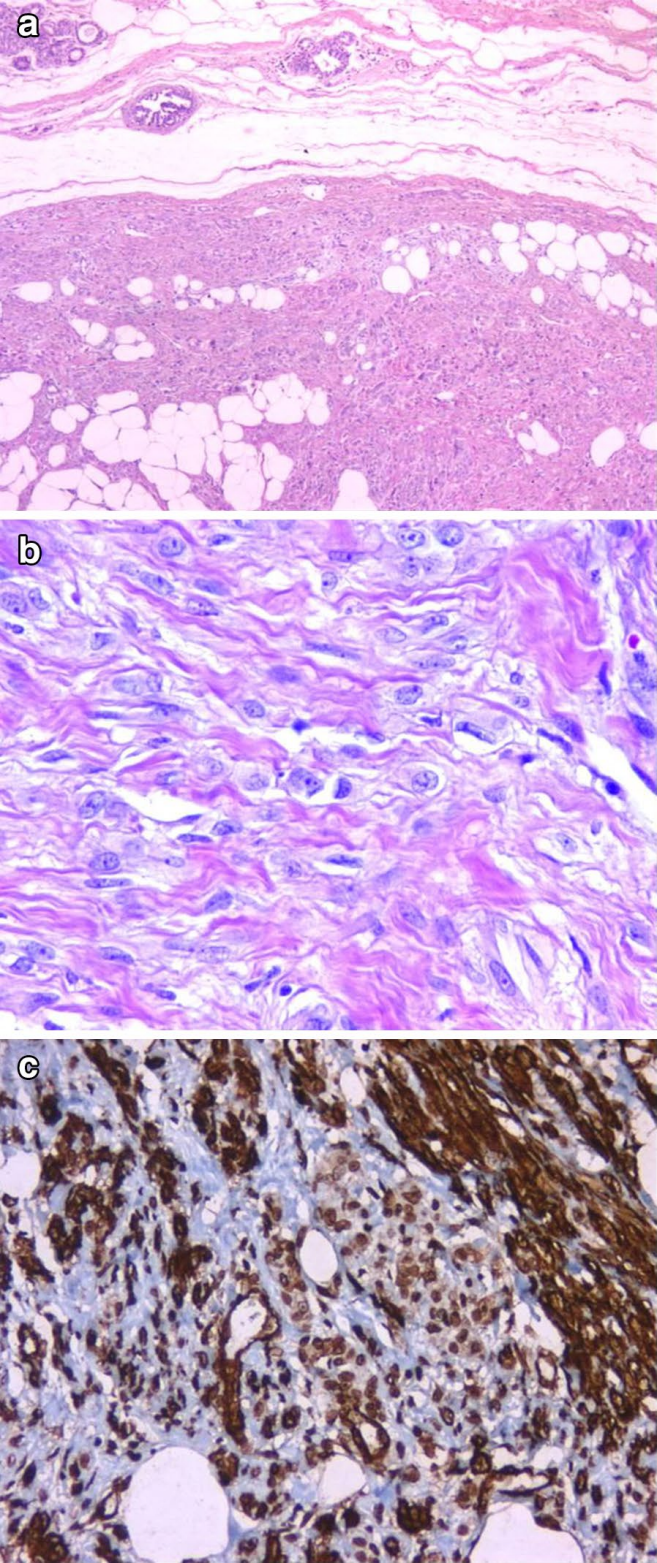
Well-circumscribed tumoral lesion consisted of fibroblasts and myofibroblasts (**a** H&Ex50; **b** H&Ex400). CD34 positivity (**c** ×200)

**Fig. 4:**
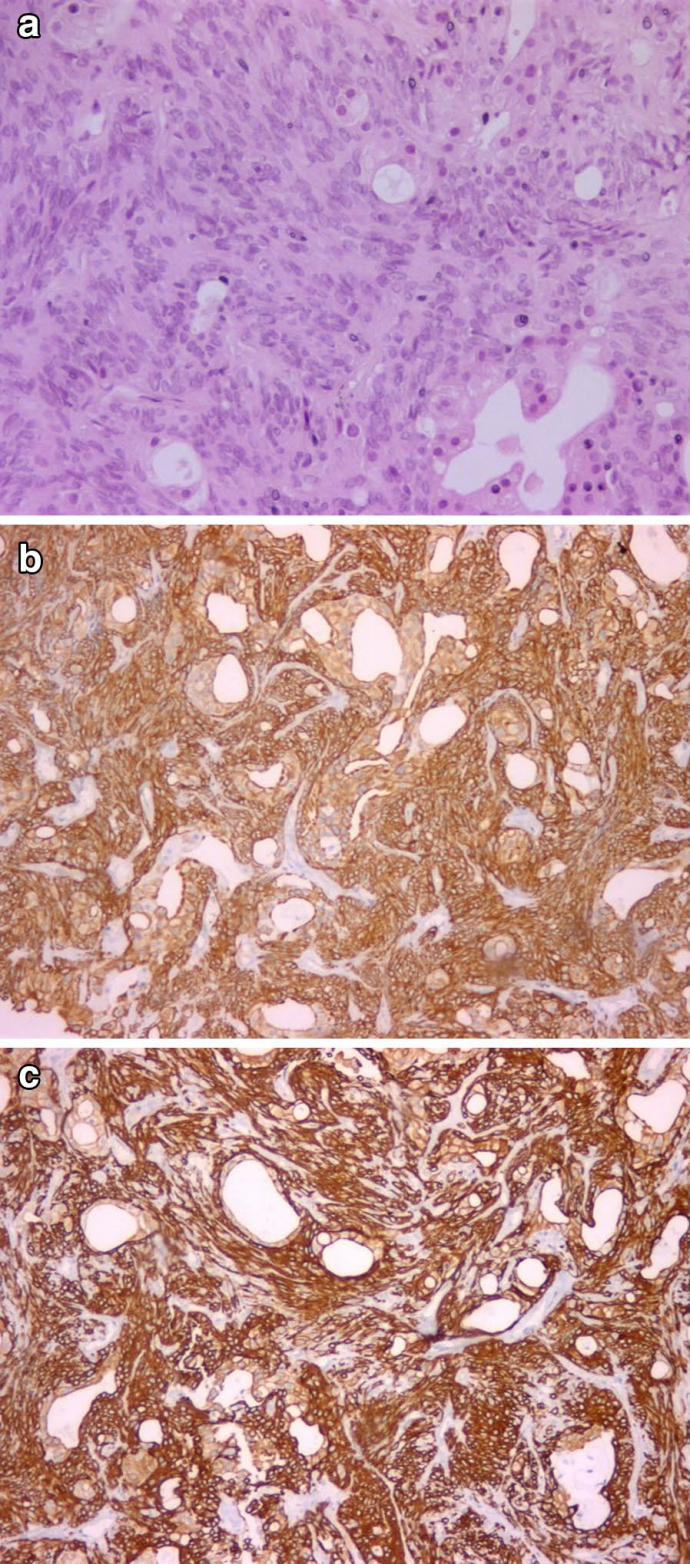
Myoepithelial cells that proliferated around epithelium-covered spaces (**a** H&Ex200), HMWK and CK14 positivity (**b**, **c** ×100)

AME: Myoepithelial cells that proliferated around epithelium-covered spaces were observed. Some of the myoepithelial cells had spindle-like characteristics (Fig. 4).

**Fig. 5:**
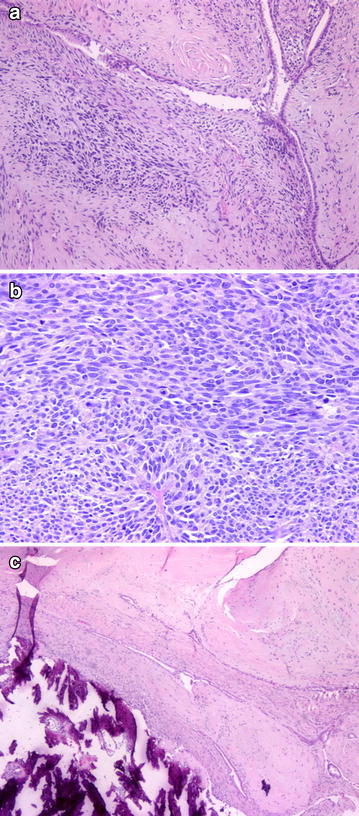
Well-circumscribed tumoral lesion consisted of epithelial cells that covered cleft-like spaces and stromal cells (**a** H&Ex100), increased mitotic activity (**b** H&Ex200), osseous metaplasia in tumoral area (**c** H&Ex50)

MF: The tumoral lesion was well-circumscribed and consisted of epithelial cells that covered cleft-like spaces and stromal cells. In the stromal cells, prominent pleomorphism, stromal overgrowth, increased mitosis (>10 per 10 hpf), and increased stromal cellularity were striking findings and also osseous metaplasia was detected (Fig. 5).

**Fig. 6:**
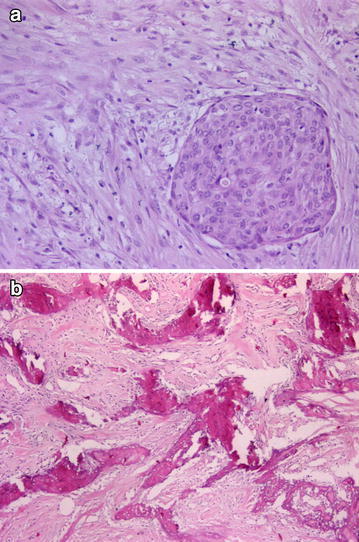
Malignant epithelial (carcinoma) and stromal (sarcoma) components of MC (**a** H&Ex200), osseous metaplasia in tumoral area (**b** H&Ex50)

MC: The tumoral lesion had an infiltrative pattern, heterogeneous appearance, and malignant epithelial (carcinoma) and stromal (sarcoma) components. Both components appeared to be distinct tumoral islands and also osseous metaplasia was detected (Fig. 6).

**Fig. 7:**
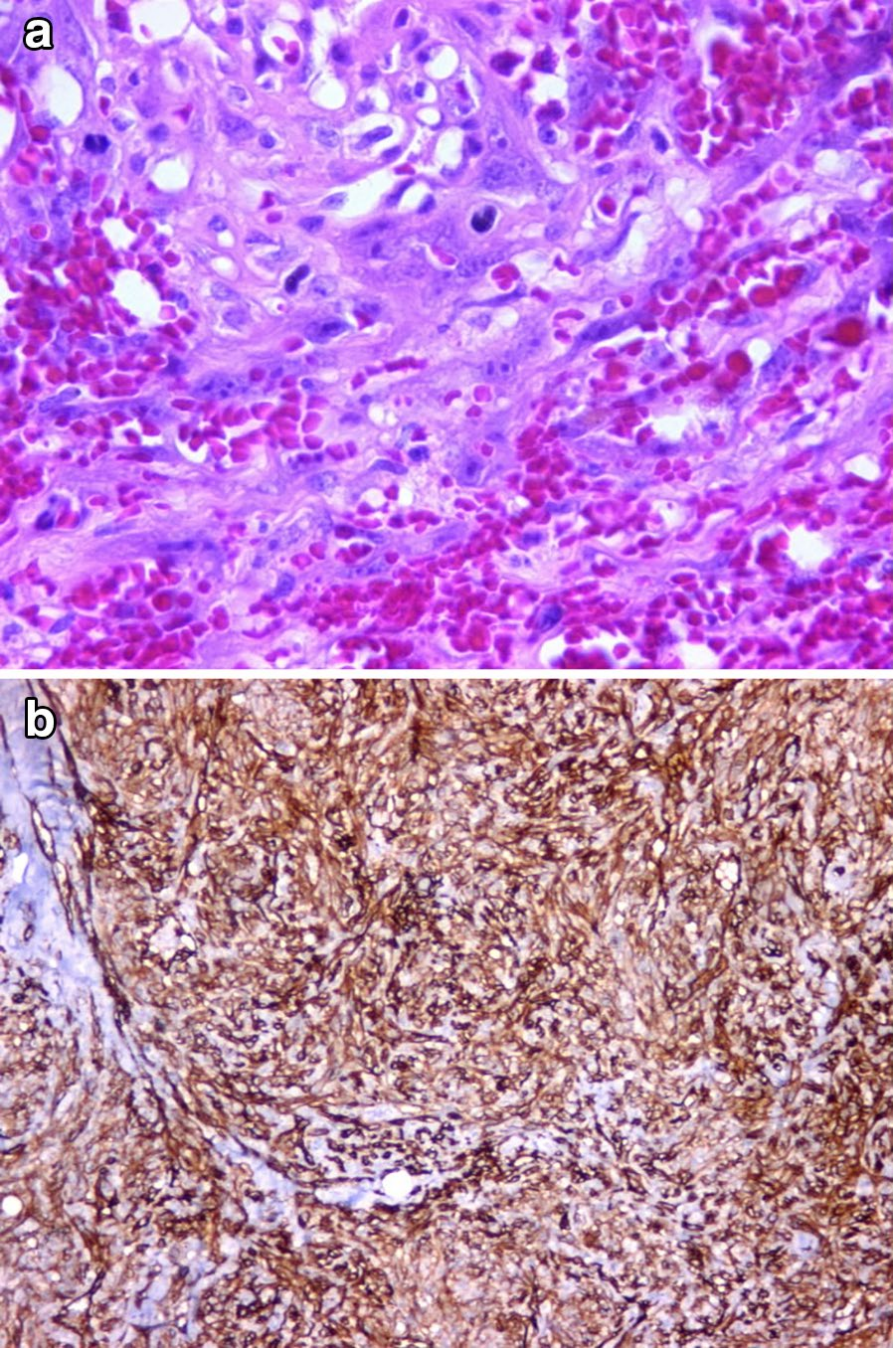
Anostomosing vascular ducts covered with pleomorphic endothelial cells (**a** H&Ex400), CD34 positivity (**b** ×100)

AS: An infiltrative tumoral lesion that was covered with pleomorphic endothelial cells was observed. Vascular ducts that anastomosed to each other were atypical, and active mitosis was detected (Fig. 7).

**Fig. 8:**
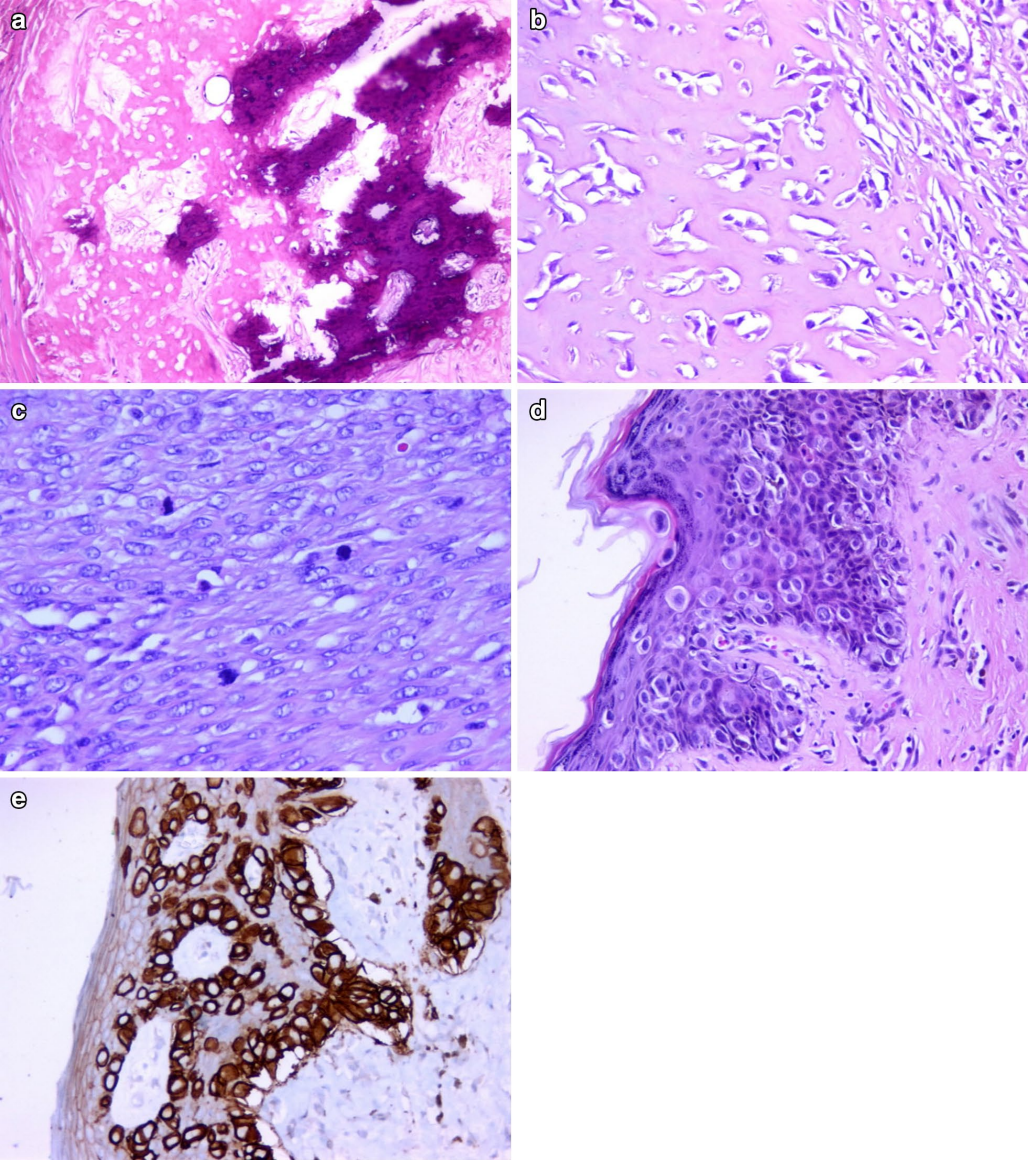
Neoplastic bone tissue (**a** H&Ex100) and lace-like osteoid material (**b** ×200). Atypical stromal cells with increased mitotic activity (**c** H&Ex400). Paget cells at overlying epidermis of nipple and CK7 positivity in these cells (**d** H&Ex200; **e** ×200)

OS: The tumor consisted of pleomorphic, spindle-shaped, ovoid, atypical cells. Osteoid and neoplastic bone tissue were seen between these cells, as well as areas with cartilage tissue. In addition, Paget cells (as single cells and in groups) with wide, pale cytoplasm and prominent nucleoli were seen in epidermis and dermis of the nipple (Fig. 8).

## Discussion

The main cause of diagnostic complexity in the approach to spindle cell lesions of the breast is the necessity of providing a correlation between clinical, radiological, morphological, and immunohistochemical findings. In addition, these lesions are rare, and it is possible to overlook them initially. On the other hand, the tumors can exhibit a biphasic pattern, such as the case of phyllodes tumors. In such cases, observing both epithelial and stromal components is diagnostic (Tan and Ellis 2013; Lakhani et al. 2012). Of course, this could be a problem for core biopsy materials. In our patient, the diagnosis was made on the core biopsy material, and this was confirmed with simple mastectomy material. Generally, in the case of phyllodes tumors, histological features, the degree of stromal hypercellularity, mitosis, and tumor boundaries are evaluated. Most PT cases are benign; recurrence might be seen in malignant forms, which are rarely seen (Tan and Ellis 2013; Tavassoli and Devilee 2003).

During the differential diagnosis, it is possible to confuse MF with stromal sarcoma; in such cases, epithelial components of MF should be examined. In addition, metaplastic carcinoma should be also considered in the differential diagnosis; in this case, the immunohistochemical staining pattern of the epithelium aids the diagnosis (Tavassoli and Devilee 2003; Stolnicu et al. 2015).

Similarly, epithelial and stromal tumor islands are seen in MCs. MCs constitute less than 1 % of all breast carcinomas, and are frequently seen in patients older than 50 years of age. Some studies have reported that the tumor diameter ranges between 3.5 and 5 cm (Lakhani et al. 2012). MCs have a heterogeneous morphology, and have malignant epithelial (carcinoma) and stromal (sarcoma) components. If the epithelial component of MC is prominent, invasive ductal carcinoma, adenosquamous carcinoma, and squamous epithelial cell carcinoma should be considered in the differential diagnosis. Squamous metaplasia is generally seen in MC, but it is not correlated with the prognosis. On the other hand, the presence of osteoid and cartilaginous metaplasia indicate a poor prognosis (Lakhani et al. 2012; Tavassoli and Devilee 2003; Wargotz and Norris 1989; Denley et al. 2000).

The other rare lesion of breast with spindle cells is AME. AME is a epithelial–myoepithelial tumour, composed of myoepithelial cells surrounding epithelium lined spaces. It comprises proliferated both two cell types (Lakhani et al. 2012).

The clinical features of patients are also crucial for the diagnostic approach. For instance, PASH is commonly encountered in premenopausal women (Ferreira et al. 2008; Wieman et al. 2008). Hormonal irregularities, gynecomastia (in males), the use of oral contraceptives, and hormone replacement therapy have been considered to play a role in its etiology (Virk and Khan 2010). PASH is a benign lesion that consists of stromal myofibroblastic cells and anastomosing vascular ducts covered with spindle cells (Lakhani et al. 2012). Hemangioma and low-grade AS should be considered in the differential diagnosis of PASH. In cases of hemangiomas, endothelial cells that cover the cleft-like vascular ducts are positive for CK and CD31. In low-grade AS, an infiltrative pattern is seen, and merging vascular ducts are covered with atypical endothelial cells that have hyper chromatic nuclei. PASH is a benign lesion, and does not contain atypia; it is possible to treat PASH with wide local excision, and CD34 positivity has diagnostic features. Myofibroblastic cells are very dense in some cases, and form bands and fascicles. It is possible to confuse these cases with myofibroblastoma. In addition, it should be discriminated from low-grade AS. In cases of AS, true vascular ducts are covered with malignant endothelial cells (Rosen 2001).

The growth pattern of lesions is especially important for a diagnostic approach. An infiltrative growth pattern is seen, particularly in malignant lesions; similarly, an infiltrative growth pattern is also seen in fibromatosis. Desmoid-type fibromatosis is a local, invasive lesion that does not have metastatic potential (Schickman et al. 2015). It originates from fibroblasts and myofibroblasts, and is usually seen in females. A history of trauma, implants, and previous surgery are predisposing factors (Lakhani et al. 2012; Jamshed et al. 2008). It is usually seen as a unilateral solitary mass. It could rarely be bilateral, just as in the current case. Prominent atypia and mitosis are not observed (Rosen 2001; Ebrahim et al. 2014). Immunohistochemical staining for CD34 is negative, and 80 % of the cases are positive for nuclear beta-catenin; however, spindle cell carcinoma, phyllodes tumors, and fibrosarcoma could also be positive. However, nodular fasciitis and scar tissue are negative for beta-catenin (Lacroix-Triki et al. 2010). The presence of macrophages in the scar tissue, foreign body-type granuloma, and presence of fat necrosis are expected in the differential diagnosis. Spindle cell carcinoma is positive for keratin and HMWK, and atypia is also present. Myofibroblastoma is positive for CD34 (Varma and Shin 2013). When discriminating from nodular fasciitis, the presence of a fast-growing, subcutaneous nodule aids the diagnosis. In addition, presence of atypia in fibrosarcoma, cellularity, CD34 positivity in phyllodes tumors, and cleft-like structures covered with epithelium are clues for the differential diagnosis.

A history of radiotherapy is another factor that could be questioned. For instance, AS ranks first among all radiation-related sarcomas (Chikarmane et al. 2014). AS is a malignant tumor that exhibits endothelial differentiation. It is considerably rare, and constitutes approximately 1 % of all soft tissue breast tumors. AS could be primary or secondary, and these are different entities. Secondary AS usually occurs secondary to radiotherapy. Primary AS is seen between the ages of 30–50, whereas secondary AS is seen in later ages (mean: 65–70) (Lakhani et al. 2012; Tavassoli and Devilee 2003). Some studies have reported the development of AS 5–10 years after radiotherapy (Hodgson et al. 2007). Primary AS develops in unradiated breast parenchyma, whereas secondary AS develops in the radiated area, in the dermal and subcutaneous tissues. Our patient, who was 28 years old, had high-grade primary AS. The patient was admitted to the clinic with pain, and had no history of radiation. AS can be divided into three groups: low (grade I), intermediate (grade II), and high (grade III). As mentioned earlier, hemangioma and PASH should be considered during the differential diagnosis of low-grade AS. High-grade AS is more common in young individuals, and shows a poor prognosis. Tumor stage is the most important prognostic factor.

Another type of sarcoma that is associated with radiotherapy is osteosarcoma. According to the literature, sarcoma development occurs 5–10 years after radiotherapy; on the other hand, our patient received radio chemotherapy for 2 years. OS constitutes 12 % of all breast sarcomas. It is important to discriminate pure osteosarcoma from malignant phyllodes tumors and heterologous osteosarcomatous differentiation of metaplastic carcinoma (Tavassoli and Eusebi 2009). In some cases, osteosarcomatous differentiation areas correspond to more than 75 % of the malignant phyllodes tumor stroma. Therefore, to achieve accurate diagnosis, a histopathological examination should be carried out with multiple sampling of the tumor tissue (Silver and Tavassoli 1998). In these lesions, the cause of admission to the clinic is usually a mass; however, patients complain of pain, tenderness, and discharge from the nipple. Similarly, our patient had pain, tenderness, and a mass.

Until today, some molecular alterations have been detected in breast lesions with spindle cell morphology. Myoepithelial differentiation may also be identified by electron microscopy (Pia-Foschini et al. 2003). In epithelial-myoepithelial tumors authors studied with 30 cases and they found 27 out of 30 were DNA diploid and the remaining 3 cases were DNA aneuploid (Fonseca and Soares 1993; Fonseca and Soares 1998). CTNNB-1 mutation and nuclear beta-catenin expression are frequently detected in sporadic breast fibromatoses, suggesting as a useful tool for differential diagnosis of breast fibromatoses from other neoplasms (Kim et al. 2012). In addition, MFB of the breast with smooth muscle differentiation showing deletion of 13q14 region (Trépant et al. 2014). And the other molecular alteration of spindle cell lesions of breast is that 70 % of MCs show EGFR gene amplification and overexpression (Rungta and Kleer 2012). So, some molecular mechanisms play role in tumor biology of breast lesions with spindle cell morphology and also Ki-67 proliferating index is the first step of immunohistochemical analysis to determine the malignant potential of the lesion.

In conclusion, cytomorphology should be considered first when approaching lesions with spindle cells. An algorithmic approach should be used in the diagnosis; cellular structure, presence and grade of atypia, growth pattern, mitotic activity, immunohistochemical staining, and clinical and radiological features should be evaluated together.
